# Regulation of Cyclooxygenase 2 by *Filifactor alocis* in Fibroblastic and Monocytic Cells

**DOI:** 10.1155/2020/4185273

**Published:** 2020-02-03

**Authors:** Marjan Nokhbehsaim, Andressa V. B. Nogueira, Sandor Nietzsche, Sigrun Eick, James Deschner

**Affiliations:** ^1^Section of Experimental Dento-Maxillo-Facial Medicine, Center of Dento-Maxillo-Facial Medicine, University of Bonn, Bonn, Germany; ^2^Department of Periodontology and Operative Dentistry, University Medical Center of the Johannes Gutenberg University, Mainz, Germany; ^3^Center of Electron Microscopy, University Hospital Jena, Jena, Germany; ^4^Department of Periodontology, Laboratory of Oral Microbiology, University of Bern, Bern, Switzerland

## Abstract

Periodontitis is a prevalent chronic inflammatory disease triggered by a synergistic and dysbiotic microbiota present in the oral biofilm. This in vitro study is aimed at evaluating the regulation of cyclooxygenase (COX)2 expression and production by the periodontopathogen *Filifactor alocis* in human gingival fibroblastic (HGF-1) and monocytic (THP-1) cells and also at investigating the underlying cellular pathway mechanisms. HGF-1 and THP-1 cells were exposed either to *F. alocis* or to the proinflammatory cytokine tumor necrosis factor alpha (TNF*α*) for 1 and 2 d to examine the COX2 expression by qPCR. COX2 protein levels were evaluated by ELISA in *F. alocis*-stimulated cells. Both types of cells were also stimulated with a blocking toll-like receptor (TLR)2 antibody or specific inhibitors against MAPKs. *F. alocis* significantly (*p* < 0.05) increased COX2 at both transcriptional and protein levels in both HGF-1 and THP-1 cells. Moreover, the stimulatory effect of *F. alocis* on COX2 was more pronounced in HGF-1 cells in comparison to THP-1 cells. *F. alocis* upregulated the COX2 expression in a dose-dependent manner in both type cells at 1 d. TNF*α* also significantly (*p* < 0.05) increased the COX2 expression in both cells. After preincubation of HGF-1 and THP-1 cells either with a neutralizing anti-TLR2 antibody or with specific MAPK inhibitors, the *F. alocis*-upregulated COX2 expression was significantly (*p* < 0.05) suppressed at 1 d. Our in vitro study provides original evidence that *F. alocis* stimulates COX2 production in fibroblastic and monocytic cells through TLR2 and MAPK mechanisms, suggesting a role of this periodontopathogen in the etiopathogenesis of periodontitis.

## 1. Introduction

Periodontitis is a chronic inflammatory disease triggered by a synergistic and dysbiotic microbiota present in the oral biofilm. Periodontopathogenic bacteria and their products activate the host immune response leading to an overproduction of proinflammatory mediators. The progression of periodontitis is associated with a sustained and excessive immune-inflammatory response resulting in irreversible destruction of the periodontal supporting tissues and, eventually, in tooth loss [1, 2]. In the periodontal tissues, both resident and infiltrating immune cells such as fibroblasts, monocytes, and macrophages are responsible for the increase in proinflammatory mediators like interleukin- (IL-) 1*β*, tumor necrosis factor alpha (TNF*α*), cyclooxygenase (COX)2, and matrix metalloproteinases. These mediators play an important role in the hard and soft tissue destruction by modulating the inflammatory cascade in periodontitis [3].

COX exists in two isoforms: COX1 and COX2. COX1 is constitutively expressed in many cells and tissues and is mainly involved in the maintenance of tissue homeostasis [4]. In contrast, COX2 is an inducible enzyme expressed by cells during inflammatory processes [4]. COX2 is induced by proinflammatory and infectious agents and is responsible for the conversion of arachidonic acid into prostaglandin (PG)E2 [5], which is actively involved in tissue damages by changing the connective tissue metabolism and by enhancing osteoclastic bone resorption, showing an evident association with the tissue destruction during periodontal disease progression [6, 7]. COX2 plays an important role in the inflammatory process in periodontal diseases. Increased levels of COX2 and PGE2 have been demonstrated in saliva, gingiva, and gingival crevicular fluid of patients with periodontal disease [8–13].

Some studies have shown a strong association between *Filifactor alocis* and periodontitis [14, 15]. This periodontopathogen is a Gram-positive anaerobic bacterium that has been detected in a higher number in saliva as well as in supragingival and subgingival biofilm of periodontal patients as compared to healthy subjects [15–18]. *F. alocis* has a high prevalence in periodontitis that could be attributed to its ability to invade periodontal cells and tissues, to resist oxidative stress and to stimulate, alone or together with periodontal pathogens, the secretion of proinflammatory cytokines [19, 20]. Although *F. alocis* has been associated with periodontitis, no previous study has examined whether this periodontopathogen modulates the expression of COX2 by professional and accessory immune cells. Thus, the aim of the present study was to evaluate the effect of *F. alocis* on the COX2 expression and production by monocytic and fibroblastic cells and to unravel the underlying pathway mechanisms.

## 2. Materials and Methods

### 2.1. Culture and Treatment of Cells

Human gingival fibroblast cell line (HGF-1) (ATCC® CRL-2014™, LGC Standards, Wesel, Germany) and human acute monocytic leukemia cell line (THP-1) (CLS Cell Lines Service, Eppelheim, Germany) were used. HGF-1 was seeded on 6-well cell culture plates (5 × 10^4^ cells/well) and grown to 80% confluence in Dulbecco's minimal essential medium (DMEM, Invitrogen, Karlsruhe, Germany) supplemented with 10% fetal bovine serum (FBS, Invitrogen), 100 units penicillin, and 100 *μ*g/ml streptomycin (Invitrogen). THP-1 was seeded once their concentration reached 1 × 10^6^ cells/ml using RPMI 1640 medium (Invitrogen) supplemented with 10% FBS, 100 units penicillin, and 100 *μ*g/ml streptomycin. Both types of cells were grown at 37°C in a humidified atmosphere of 5% CO_2_, and every other day, the cell culture medium was changed. One day prior to the experiments, the FBS concentration was reduced to 1%.

The oral pathogenic bacterium *F. alocis* ATCC 35896™ was used to stimulate the cells at different concentrations (optical density (OD): 0.05, 0.1, and 0.2) as in our previous study [21]. *F. alocis* was precultivated in an anaerobic atmosphere on Schaedler agar plates (Oxoid, Basingstoke, UK) for 48 h and, then, suspended in phosphate-buffered saline (OD_660nm_ = 1, equivalent to 1.2 × 10^9^ bacterial cells/ml). Subsequently, the bacteria suspension was exposed twice to ultrasonication (160 W for 15 min) resulting in total bacterial killing. In order to unravel intracellular signaling mechanisms by which *F. alocis* could possibly modulate COX2 expression, cells were preincubated with specific inhibitors of different pathways (MEK1 and MEK2: U0126, 10 *μ*M; P38: SB203580, 10 *μ*M; pyrrolidine dithiocarbamate: PDTC, 10 *μ*M; and JNK inhibitor II: SP600125, 10 *μ*M; all from Calbiochem, San Diego, CA, USA), 1 h prior to the *F. alocis* stimulation. Furthermore, cells were also preincubated with a blocking anti-human toll-like receptor (TLR) 2 monoclonal antibody (10 *μ*g/ml; eBioscience, San Diego, CA, USA) 45 min prior to the *F. alocis* stimulation. Moreover, in order to mimic inflammatory conditions in vitro, cells were treated with human recombinant tumor necrosis factor (TNF)*α* (Biomol, Hamburg, Germany), which is one of the key inflammatory mediators in the pathogenesis of the periodontal diseases. TNF*α* was applied at a concentration of 1 ng/ml, which is in the range of levels usually found in the gingival crevicular fluid (GCF) of periodontally diseased patients and which has been used by other investigators [22, 23]. Untreated cells served as a control.

### 2.2. Scanning Electron Microscopy

Scanning electron microscopy images were taken to visualize the attachment of *F. alocis* ATCC 35896 on fibroblasts. The cells were cultured on glass slides until a monolayer was formed and thereafter exposed to *F. alocis* for 1 h. Samples were fixed with 2% glutaraldehyde in 0.1 M cacodylate buffer for 30 min, washed twice with cacodylate buffer, and dehydrated using a 10% graded ethanol series (10 min each concentration). At the Center of Electron Microscopy, University Hospital of Jena, Germany, critical point drying was performed, and samples were sputter-coated with gold and examined with a ZEISS LEO-1530 Gemini (Carl Zeiss NTS, Oberkochen, Germany) equipped with a field emission electron gun at 10 keV.

### 2.3. Real-Time PCR

Total RNA was extracted by using a RNA extraction kit (RNeasy Mini Kit, Qiagen, Hilden, Germany) following the manufacturer's protocol. Subsequently, RNA concentration was verified by a spectrophotometer (NanoDrop ND-2000, Thermo Fisher Scientific, Wilmington, DE, USA), and 500 ng of total RNA was transcribed to cDNA using the iScript™ Select cDNA Synthesis Kit (Bio-Rad Laboratories, Munich, Germany) at 42°C for 90 min followed by 85°C for 5 min, according to the manufacturer's instructions. The gene expression of COX2 and glyceraldehyde-3-phosphate dehydrogenase (GAPDH) as an endogenous control was evaluated by quantitative RT-PCR by using an iCycler iQ5 Real-Time PCR Detection System (Bio-Rad). A 25 *μ*l PCR reaction mixture was prepared containing 1 *μ*l of cDNA, 12.5 *μ*l of SsoAdvanced™ Universal SYBR® Green Supermix (Bio-Rad), 2.5 *μ*l of primers, and 9 *μ*l RNase-free water. The thermal protocol of the PCR reaction was 95°C for 5 min, followed by 40 cycles of denaturation at 95°C for 10 s and combined annealing/extension at 60°C for 30 s. This analysis was performed in triplicate. The data analysis of gene expression was performed using the comparative threshold cycle (CT) method.

### 2.4. ELISA

HGF-1 or THP-1 cell lysates were used to evaluate the levels of COX2 by using commercially available sandwich enzyme-linked immunoassay (ELISA) detection kit (DYC4198-2; human/mouse Total COX2 ELISA with DuoSet, R&D Systems Europe, Abingdon, United Kingdom) according to the manufacturer's instructions. The COX2 concentration was measured by spectrophotometry using a microplate reader (PowerWave X, BioTek Instruments, Winooski, VT, USA) at 450 nm with wavelength correction at 540 nm. Total protein concentration was measured using Pierce BCA Protein Assay Kit (23227, Thermo Fisher Scientific, Pierce Biotechnology, Rockford, USA) and used to normalize the data.

### 2.5. Statistical Analysis

Statistical analysis was performed using the software IBM SPSS Statistics 22. The mean values and standard errors of the mean (SEM) were calculated. For statistical analysis, parametric (ANOVA followed by post hoc Dunnett's and Tukey's tests) and nonparametric tests (Wilcoxon and Mann-Whitney *U* tests) were used. Significant differences between groups were considered at *p* value < 0.05. All experiments were performed in triplicate and repeated at least twice.

## 3. Results

### 3.1. *F. alocis* Accumulation on Gingival Fibroblast

**Figure 1:**
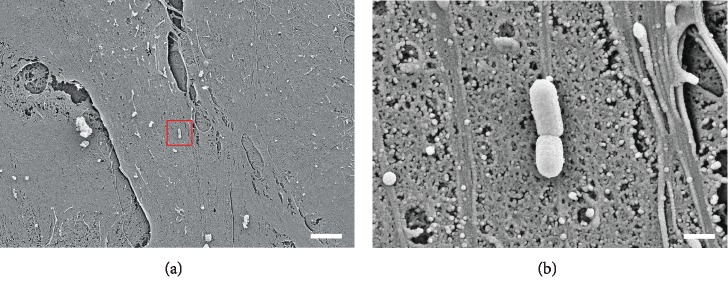
(a, b) Scanning electron microscopy images of *F. alocis* attached to fibroblastic cells. Scale bars: 5 *μ*m (a) and 500 nm (b).

**Figure 2:**
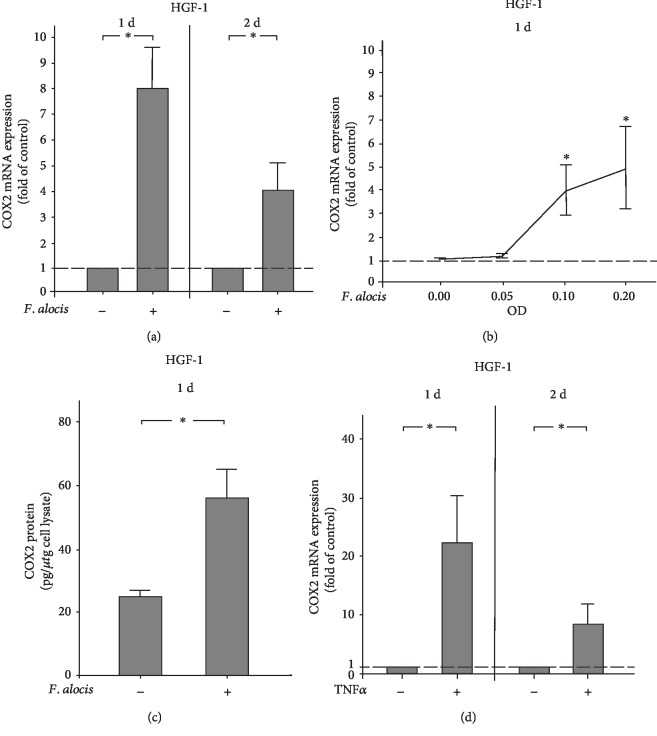
(a) Expression of COX2 in the presence and absence of *F. alocis* (OD_660_: 0.1) in HGF-1 cells at 1 d and 2 d, as analyzed by qPCR. Mean ± SEM (*n* = 18). ^∗^Significant (*p* < 0.05) difference between groups. (b) Stimulation of COX2 expression by various concentrations of *F. alocis* (OD_660_: 0.05, 0.1, and 0.2) in HGF-1 cells at 1 d, as analyzed by qPCR. Unstimulated cells served as the control. Mean ± SEM (*n* = 6). ^∗^Significantly (*p* < 0.05) different from control. (c) COX2 protein level in lysates of HGF-1 cells in the presence and absence of *F. alocis* (OD_660_: 0.1) at 1 d, as analyzed by ELISA. Mean ± SEM (*n* = 12). ^∗^Significant (*p* < 0.05) difference between groups. (d) Expression of COX2 in the presence and absence of TNF*α* (1 ng/ml) in HGF-1 cells at 1 d and 2 d, as analyzed by qPCR. Mean ± SEM (*n* = 26). ^∗^Significant (*p* < 0.05) difference between groups.

Figures 1(a) and 2(b) show *F. alocis* and its capability to attach to periodontal fibroblastic cells.

### 3.2. Regulation of COX2 by *F. alocis* in HGF-1 Cells

First, we investigated the regulation of COX2 in the presence and absence of the periodontopathogen *F. alocis* in HGF-1 cells. As shown in Figure 2(a), *F. alocis* caused a significant (*p* < 0.05) increase in the constitutive COX2 expression at 1 and 2 d, as analyzed by qPCR. Furthermore, the stimulatory effect of *F. alocis* on the COX2 expression was dose-dependent at d, with the highest COX2 expression at the highest concentration of *F. alocis* (Figure 2(b)). The stimulatory action of *F. alocis* on COX2 was also found at the protein level at 1 d. COX2 protein levels were significantly (*p* < 0.05) higher in cell lysates from *F. alocis*-stimulated HGF-1 cells as compared to unstimulated control cells, as determined by ELISA (Figure 2(c)). Interestingly, the proinflammatory cytokine TNF*α* also significantly (*p* < 0.05) increased the COX2 expression in HGF-1 cells at 1 d and 2 d (Figure 2(d)).

### 3.3. Regulation of COX2 by *F. alocis* in THP-1 Cells

**Figure 3:**
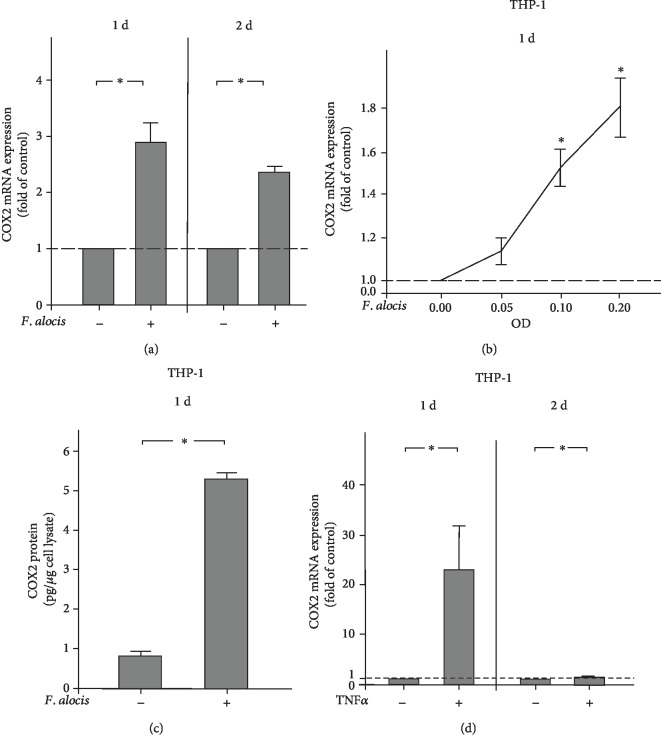
(a) Expression of COX2 in the presence and absence of *F. alocis* (OD_660_: 0.1) in THP-1 cells at 1 d and 2 d, as analyzed by qPCR. Mean ± SEM (*n* = 18). ^∗^Significant (*p* < 0.05) difference between groups. (b) Stimulation of COX2 expression by various concentrations of *F. alocis* (OD_660_: 0.05, 0.1, and 0.2) in THP-1 cells at 1 d, as analyzed by qPCR. Unstimulated cells served as the control. Mean ± SEM (*n* = 18). ^∗^Significantly (*p* < 0.05) different from the control. (c) COX2 protein level in lysates of THP-1 cells in the presence and absence of *F. alocis* (OD_660_: 0.1) at 1 d, as analyzed by ELISA. Mean ± SEM (*n* = 18). ^∗^Significant (*p* < 0.05) difference between groups. (d) Expression of COX2 in the presence and absence of TNF*α* (1 ng/ml) in THP-1 cells at 1 d and 2 d, as analyzed by qPCR. Mean ± SEM (*n* = 12). ^∗^Significant (*p* < 0.05) difference between groups.

Next, we sought to evaluate the influence of *F. alocis* on COX2 expression and protein levels in THP-1 cells. Like in HGF-1 cells, *F. alocis* caused a significant (*p* < 0.05) upregulation of COX2 in THP-1 cells at 1 d and 2 d, as demonstrated in Figure 3(a). Moreover, the upregulation of COX2 expression by *F. alocis* was also dose-dependent in THP-1 cells. Again, the highest COX2 expression was observed at the highest concentration of *F. alocis*, and the protein levels were also paralleled by the gene expression levels of COX2 (Figures 3(b) and 3(c)). Following stimulation with TNF*α*, THP-1 cells showed a significantly (*p* < 0.05) increased COX2 expression at 1 d but not 2 d (Figure 3(d)).

### 3.4. Involvement of TLR2 and Signaling Pathways in the Actions of *F. alocis*

**Figure 4:**
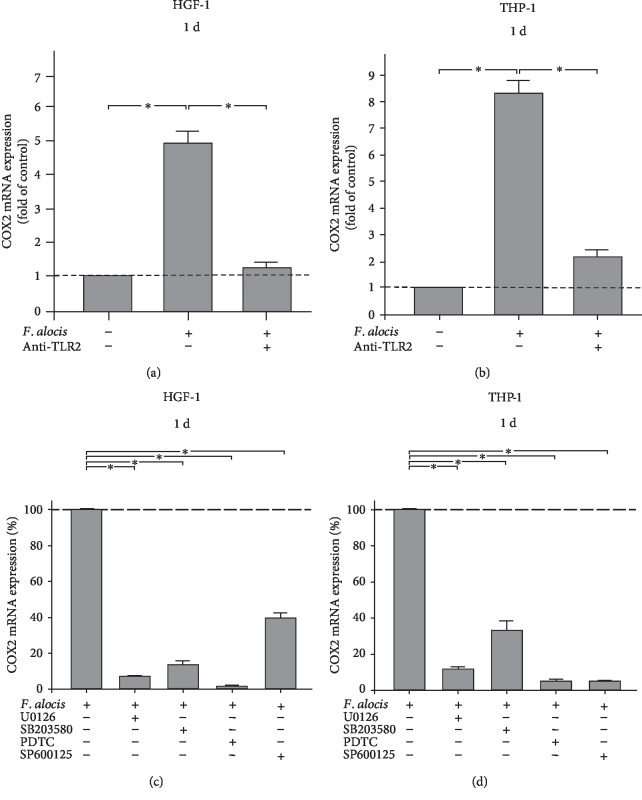
COX2 gene expression in response to *F. alocis* (OD_660_: 0.1) in the presence and absence of anti-TLR2 blocking antibody in HGF-1 (a) and THP-1 (b) cells at 1 d, as analyzed by qPCR. Mean ± SEM (*n* = 3). ^∗^Significant (*p* < 0.05) difference between groups. COX2 gene expression in response to *F. alocis* (OD_660_: 0.1) in the presence and absence of specific inhibitors against MEK1/MEK2 (U0126; 10 *μ*M), p38 (SB203580; 10 *μ*M), NF-*κ*B (PDTC; 10 *μ*M), and JNK (SP600125; 10 *μ*M) in HGF-1 (c) and THP-1 (d) cells at 1 d, as analyzed by qPCR. Mean ± SEM (*n* = 3). ^∗^Significant (*p* < 0.05) difference between groups.

Next, we sought to elucidate whether the stimulatory effects of *F. alocis* on COX2 expression were mediated through TLR2. Interestingly, when HGF-1 and THP-1 cells were preincubated with an anti-TLR2 blocking antibody, the stimulatory effect of *F. alocis* on COX2 was significantly (*p* < 0.05) abolished at 1 d, as shown in Figures 4(a) and 4(b). Furthermore, we examined the involvement of intracellular pathways typically associated with inflammatory processes. HGF-1 and THP-1 cells were preincubated with specific inhibitors for MEK1/MEK2 (U0126), p38 (SB203580), NF-*κ*B (PDTC), and JNK (SP600125) signaling, which resulted in a significant (*p* < 0.05) reduction of the *F. alocis*-induced COX2 expression for each of these inhibitors at 1 d (Figures 4(c) and 4(d)).

## 4. Discussion

Our study provides original evidence that the putative oral pathogen *F. alocis* is capable of inducing COX2 at transcriptional and protein levels in human fibroblastic and monocytic cells through TLR2 and MAPK signaling, suggesting a role of *F. alocis* in the etiopathogenesis of periodontal diseases. Interestingly, the stimulatory effect of *F. alocis* on COX2 was more pronounced in fibroblasts as compared to the monocytic cells, which emphasizes the significance of periodontal fibroblasts as accessory inflammatory cells in periodontal inflammation.

The human oral microbiome comprises of more than 700 prokaryote species, and one of the periodontopathogens is *F. alocis* [24]. *F. alocis* is a Gram-positive, asaccharolytic, obligate anaerobic microorganism with a high prevalence in periodontal diseases [25]. Due to its virulence factors, *F. alocis* has the capacity to resist to oxidative stress, form biofilms, interact with other periodontopathogens, and produce proteases. Furthermore, periodontopathogens, such as *F. alocis*, are able to invade gingival cells and tissues, as the pocket epithelium is ulcerated in periodontitis, and can therefore get into the subepithelial blood vessels. As a consequence, infections with *F. alocis* result in the activation of the host immune response and, subsequently, the release of proinflammatory mediators by immunoinflammatory cells [19, 20, 26]. Ultimately, the dysregulated and excessive immunoinflammatory processes lead to soft tissue damage and alveolar bone resorption. COX2 is a key molecule of periodontal inflammation. Increased COX2 levels were found at sites of gingival or periodontal inflammation, and its expression has been shown to be associated with the severity of the disease [10, 11, 27]. High COX2 levels in inflamed gingival tissues leads, in turn, to elevated PGE2 levels, which then further mediates the soft and hard tissue destruction [28], which highlights the importance of COX2 in the etiopathogenesis of periodontitis. Consequently, COX2 inhibitors have been evaluated for their adjunctive benefit in the treatment of periodontal diseases [29–31]. To date, no study has focused on the effect of *F. alocis* on the COX2 regulation, which could mediate, at least in part, the pathogenic actions of this microorganism on periodontal tissues. In this context, we sought to analyze what role structural resident cells of the periodontium, e.g., gingival fibroblasts, play as compared to professional inflammatory cells, e.g., monocytic cells.

Our data demonstrates for the first time that *F. alocis* upregulates the COX2 expression and increases the protein synthesis in both HGF-1 and THP-1 cells, suggesting that this putative pathogen also contributes to the elevated levels of COX2 in gingival tissues of periodontitis patients. Remarkably, the fibroblastic cells showed a more pronounced inflammatory response to *F. alocis* as compared to the monocytic cells, demonstrating the critical role of periodontal cells in the etiopathogenesis of periodontal diseases. It would be very interesting to also examine the effect of *F. alocis* on COX2 in gingival epithelial cells in future studies. Interestingly, other investigators have observed that IL-1*β* and *Aggregatibacter actinomycetemcomitans* as well as *Tannerella forsythia* stimulate the COX2 expression in gingival fibroblasts, which is in agreement with our findings for TNF*α* and *F. alocis* [28]. We have previously demonstrated that *Fusobacterium nucleatum* is able to modulate COX2 levels in human periodontal ligament fibroblasts [32]. In summary, these observations suggest that periodontopathogens may use COX2 for their detrimental effects on periodontal cells and tissues.

Preincubation with a blocking TLR2 antibody resulted in a significant inhibition of the *F. alocis*-induced COX2 expression in HGF-1 and THP-1 cells, suggesting that *F. alocis* is interacting with this TLR for its inflammatory actions. Also, preincubation of HGF-1 and THP-1 cells with specific blockers against MAPK signaling, such as MEK1/MEK2, p38 MAPK, NF-*κ*B, and JNK, caused an inhibition of the *F. alocis*-upregulated COX2 expression, indicating that these signaling pathways are involved in the regulation of COX2 by *F. alocis* in fibroblastic and monocytic cells. Recently, it has been demonstrated that interaction of *F. alocis* with neutrophils through TLR2 and subsequent activation of ERK1/2 and p38 MAPK pathways results in granule exocytosis as well as random and directed migration [16], which is in accordance with the observation that *F. alocis* exploits TLR2 and MAPKs for its effects. However, additional pathways could be involved in the actions of *F. alocis* on COX2 and should be studied in further investigations. In our experiments, a lysate of *F. alocis* was applied and the protease activity was still maintained. The used concentration of the bacterial lysate was also applied in previous studies on other periodontopathogens [32–34]. Although the knowledge on the *F. alocis* cell wall components is still limited, it could be assumed that lipoteichoic acid and/or peptidoglycan, which are present on the cell wall of Gram-positive bacteria, can directly stimulate fibroblasts or monocytes. Additional virulence factors of *F. alocis* could also have been contributed to the stimulatory effects of this microorganism on COX2 in our cells. Therefore, other receptors in addition to TLR2 may also have been implicated in the COX2 regulation.

In order to better understand the pathogenic role of *F. alocis* in the etiopathogenesis of periodontal diseases, this periodontopathogen was selected for our study. The classical periodontopathogens *P. gingivalis and F. nucleatum* were also shown to upregulate COX2, which was used as a target in our study, in immunoinflammatory and periodontal fibroblastic cells, emphasizing again the key role of COX2 in the pathogenesis of periodontitis [32, 35]. Since periodontitis is a polymicrobial disease, the effect of *F. alocis* in combination with other periodontopathogens should also be considered in future studies.

In one of our previous and recent study, we have used similar methodology but different experiments to evaluate the effect of *F. alocis* on the regulation of the collagenase MMP-1 in HGF-1 and THP-1 cells. We have demonstrated for the first time that *F. alocis* significantly increases the expression and protein synthesis of MMP-1 in both HGF-1 and THP-1 cells [21]. Interestingly, our results show original evidence that *F. alocis* may contribute to periodontal destruction through MMP-1 and to periodontal inflammation through COX2, demonstrating its role in two different stages of the periodontal disease process.

In summary, our in vitro study provides novel evidence that *F. alocis* upregulates COX2 at both transcriptional and protein levels through TLR2 and MAPK signaling mechanisms in human gingival fibroblastic and monocytic cells, suggesting that *F. alocis* may play a role in periodontal disease initiation and progression, therefore contributing to periodontal inflammation and tissue destruction. Moreover, the stimulatory action of *F. alocis* on COX2 was more pronounced in gingival fibroblasts as compared to the monocytic cells, underlining the critical role of these fibroblasts as accessory inflammatory cells in periodontal inflammation.

## Data Availability

The data used to support the findings of this study are available from the corresponding author upon request.
